# Back to past leeches: repeated phlebotomies and cardiovascular risk

**DOI:** 10.1186/1741-7015-10-53

**Published:** 2012-05-30

**Authors:** Melania Manco, Josè Manuel Fernandez-Real

**Affiliations:** 1 Bambino Gesù Children's Hospital and Research Institute, Research Unit for Multifactorial Disease. Piazza San Onofrio 4, 00165, Rome. Italy; 2 University Hospital of Girona 'Dr Josep Trueta', Carretera de França s/n, 17007, Girona, Spain; 3 Department of Diabetes, Endocrinology and Nutrition, Institut d'Investigació Biomédica de Girona IdibGi, Girona, Spain; 4 CIBER Fisiopatología de la Obesidad y Nutrición. Hospital of Girona 'Dr Josep Trueta', Carretera de França s/n, 17007, Girona, Spain

**Keywords:** blood pressure, insulin sensitivity, iron, metabolic syndrome, phlebotomy

## Abstract

In patients with metabolic syndrome, body iron overload exacerbates insulin resistance, impairment of glucose metabolism, endothelium dysfunction and coronary artery responses. Conversely, iron depletion is effective to ameliorate glucose metabolism and dysfunctional endothelium. Most of its effectiveness seems to occur through the amelioration of systemic and hepatic insulin resistance.

In a study published by *BMC Medicine*, Michalsen *et al. *demonstrated a dramatic improvement of blood pressure, serum glucose and lipids after removing 550 to 800 ml of blood in subjects with metabolic syndrome. This effect was apparently independent of changes in insulin resistance, in contrast to previous cross-sectional and cohort studies investigating the association between iron overload, insulin resistance and cardiovascular disease.

Despite drawbacks in the study design, its findings may lead the way to investigations aimed at exploring iron-dependent regulatory mechanisms of vascular tone in healthy individuals and patients with metabolic disease, thus providing a rationale for novel preventive and therapeutic strategies to counteract hypertension.

Please see related article: http://www.biomedcentral.com/1741-7015/10/54

## Background

Metabolic syndrome (MetS) is a clustering of cardiovascular disease risk factors (for example, visceral, obesity dyslipidemia, hypertension, hyperglycemia, fatty liver disease). Both insulin resistance (IR) and low-grade inflammation underlie the syndrome [1]. The presence of MetS is predictive of type 2 diabetes mellitus (T2DM) and all-cause mortality [2]. In the United States, prevalence estimates of MetS are 33% for the general younger adult population (aged 20 to 59) and 59% in older individuals. In the future, metabolic syndrome may overtake smoking as the leading risk factor for heart disease [3]. Currently, it is possible to prevent or delay the occurrence of MetS, mainly with a healthy lifestyle, which is a lifelong commitment [1].

Other strategies are also possible. Michalsen *et al. *[4] observed an impressive reduction of blood pressure (about 18 mmHg in the treated group compared with 0.2 in the control group) in patients with MetS after removing 550 to 800 ml of blood. The effect was evident early after the first phlebotomy and persisted two weeks following the second venopuncture (performed at Day 28). The study was a randomized, controlled single-blinded trial of 64 hypertensive patients. Thirty-seven percent had T2DM. Most of them were on drug treatment. Changes in blood pressure and insulin resistance (as estimated by the homeostatic model assessment of insulin resistance, HOMA-IR) correlated with the reduction in ferritin levels.

The beneficial effects of phlebotomies have been repeatedly observed in the past and documented in 1867 by Brunton, who observed that "Small bleedings of three or four ounces, whether by cupping or venesection, were (...) beneficial", and recommended that a few ounces of blood be removed every few weeks from patients with angina. He attributed the relief of angina to the diminution of "arterial tension" [5]. In 1970, Parker *et al. *[6] observed that angina was relieved and ventricular function returned to normal after a phlebotomy averaging 276 ml. More recently, iron overload was observed to cluster with abnormalities of MetS, including overt T2DM [7-9]. Iron depletion by repeated phlebotomies, erythrocytapheresis or use of iron chelators ameliorated metabolic control, coronary artery responses and endothelial dysfunction [7-9] and the beneficial effects of these procedures on metabolism are thought to be driven primarily by the amelioration of insulin resistance [9,10].

Puzzlingly, the beneficial effect on blood pressure observed by Michalsen [4] was independent of any effect on IR; hence, suggesting an independent mechanism of action. The potential impact of the Michalsen's study is remarkable in terms of reduced health care costs. If effectiveness of repeated phlebotomies in reducing blood pressure in patients with MetS is confirmed, this could dramatically reduce the burden of the syndrome and its related costs. Indeed, phlebotomies as routine means for primary prevention or treatment of hypertension are easy to perform, low-in-cost and, hence, could be a good alternative to more expensive drugs. Moreover, by encouraging blood donation in patients with MetS it will be effective also to respond to the worldwide significant demand for red cells and blood components.

However, the results from this trial generate a number of questions regarding the lack of effect on IR and the early and consistent effects on blood pressure.

### Iron overload, insulin resistance and risk of T2DM

A relationship between iron stores and IR has been reported, but with some inconsistencies. The risk of diabetes was significantly lower in frequent blood donors from the Health Professionals Follow-up Study when compared with non-donors after 10 years of follow-up [11]. However, the beneficial effect of frequent blood donation disappeared at 12 years [12]. In the same cohort [12] and in post menopausal women from the Iowa Women's Health Study [13], heme-iron intake from red meat sources was associated with increased risk of diabetes.

Serum ferritin, a surrogate marker of iron status as it accurately reflects body iron stores in healthy individuals [14], was associated with increased risk of T2DM [15]. Subjects with hyperferritinemia had a 2.4-fold higher risk of developing T2DM [16]. This association was further confirmed in other studies [17-19].

None of these aforementioned studies provided information on insulin resistance, which has been explored in cross-sectional investigations. Frequent blood donors had improved insulin sensitivity and decreased insulin secretion when compared with sporadic blood donors or non-donors [20]. In an unblinded and uncontrolled study of 31 subjects with glucose intolerance, serial phlebotomy individually adjusted to induce near iron deficiency was associated with improved insulin sensitivity in response to oral glucose loading and reduced levels of glycated haemoglobin [21]. In another unblinded, randomized study in 28 patients with high-ferritin T2DM, three serial 500-ml phlebotomies reduced mean serum ferritin from 460 to 232 ng/ml and simultaneously led to increased insulin sensitivity and reduced levels of glycated haemoglobin [8].

Concerning the relationship between iron stores, MetS and IR, a number of epidemiological studies showed a relationship [22-24]. Hence, the lack of a significant effect of phlebotomies on IR in the present series is somewhat unexpected. HOMA is a poor predictor of insulin sensitivity in patients with T2DM, but, additionally, more than two phlebotomies and steadily reduced ferritin levels might be needed to see a significant effect of iron depletion on IR in patients with long-term diabetes and on multi-drug treatment as those evaluated in the Michalsen's series [4]. Indeed, significant effects after one year of treatment in high-ferritin T2DM patients [7] and after two years in those carrying hereditary hemochromatosis [10] have been observed. Moreover, drug treatment might have confounded results and thus, it would be important to study in more depth the effects on HOMA-IR by taking into account the use of medication (that is, separating those taking metformin vs. the other groups). Alternatively, the effects of blood donation on IR may differ in subjects with normal versus abnormal glucose tolerance as observed when exploring the effect of iron depletion on brachial artery flow-mediated dilation [25] and in keeping with those of Hirai *et al. *[26], who demonstrated differential effects of vitamin C in subjects with normal versus abnormal glucose tolerance.

### Behind the iron-heart hypothesis

Several epidemiological studies have investigated the effect of iron depletion on atherosclerosis, cardiovascular risk, mortality and morbidity [27]. Blood pressure is one of the main factors driving cardiovascular morbidity and mortality, but no evidence was provided of any reduction in blood pressure following phlebotomies.

By stratifying men from the Health Professionals Follow-up Study according to lifetime number of donations (0, 10 to 20 and ≥30), no difference was observed in the risk for hypertension [11]. The Kuopio Study was the study which reported significant differences in mean blood pressures between donors and non-donors, but such a difference might reflect different lifestyles [16].

Thus, one of the main questions is how two phlebotomies worked to reduce blood pressure. Improvement of IR is not a nominee, at least at a first glance. Michalsen *et al. *[4] argue that reduced low-grade inflammation and oxidative stress may play a role as iron-dependent hydroxyl radical formation can contribute to vascular dysfunction. In this regard, high-frequency blood donation was associated with reduced serum ferritin and increased brachial artery flow-mediated dilation compared with low-frequency blood donation [22]. Serum ferritin was significantly decreased and flow-mediated dilation was increased in high-frequency donors compared with low-frequency donors with no between-group difference in insulin sensitivity. The decrease in flow-mediated dilation during oral glucose tolerance testing did not differ between high- and low-frequency blood donors [22]. Alternatively, the authors [4] speculate that changes in blood viscosity caused vasodilatation and, in turn, reduced blood pressure.

On the other hand, as blood volume was not replaced following phlebotomies, patients on multi-drug therapy or with type 2 diabetes might also have a dysfunctional endothelium and sympathetic response to relative hypovolemia. They might be unable to compensate for hypovolemia as healthy donors do. Indeed, most previous evidence were from cohort and cross-sectional studies of healthy donors [20], and from high-ferritin T2DM patients and carriers of hereditary hemocromatosis in whom blood volume was restored to normal at each procedure [7,10].

### Future directions and conclusions

The strength of Michalsen's study is that for the first time an effect of iron depletion on blood pressure is recognized. Like other evidence from clinical studies, the effectiveness of iron depletion on hypertension needs to be proved with further studies. The focus of the research should move toward two different fronts: i) investigation of the effect of reducing body iron stores through graded phlebotomy, preferably using solid measures of insulin sensitivity, vascular resistance, viscosity and oxidative damage; ii) meta-analysis of data from published cohorts or *de novo *analyses of much larger cohorts of healthy donors and patients with an appropriately long-term follow-up and tight monitoring of dietary iron intake.

In conclusion, findings from Michalsen's study give new perspectives for prevention and treatment of the metabolic syndrome demonstrating that repeated phlebotomies, a low-cost and minimally invasive technique, are effective in reducing blood pressure with a mechanism that is independent of insulin resistance. Routine phlebotomies in these patients may enormously reduce health care costs related to the epidemic metabolic syndrome and, importantly, also contribute to increase the rate of blood donations.

## Abbreviations

HOMA: homeostatic model assessment; IR: insulin resistance; MetS: metabolic syndrome; T2DM: type 2 diabetes mellitus.

## Competing interests

The authors have no conflict of interest in relation to this work.

## Authors' contributions

Both authors contributed equally to the drafting and revising of the manuscript, and both authors approved the final version to be published.

## Authors' information

MM, MD Ph.D FACN, is responsible for the research unit for multifactorial diseases at the Scientific Directorate of the Bambino Gesù Children's Hospital, Rome. She has devoted her entire research activity to obesity and type 2 diabetes.

JMFR, MD, PhD, is Chief of the Section of Diabetes. Department of Endocrinology. Hospital de Girona Dr Josep Trueta and CIBERobn. Avinguda de França s/n. 17007 Girona, Spain.

## Pre-publication history

The pre-publication history for this paper can be accessed here:

http://www.biomedcentral.com/1741-7015/10/53/prepub
